# Curved Walking Rehabilitation with a Rotating Treadmill in Patients with Parkinson’s Disease: A Proof of Concept

**DOI:** 10.3389/fneur.2017.00053

**Published:** 2017-02-28

**Authors:** Marco Godi, Marica Giardini, Antonio Nardone, Anna Maria Turcato, Marco Caligari, Fabrizio Pisano, Marco Schieppati

**Affiliations:** ^1^Istituti Clinici Scientifici Maugeri Spa SB, IRCCS, Division of Physical Medicine and Rehabilitation, Scientific Institute of Veruno, Veruno, Italy; ^2^Department of Translational Medicine, University of Eastern Piedmont, Novara, Italy; ^3^Istituti Clinici Scientifici Maugeri Spa SB, IRCCS, Laboratorio di Comunicazione e Domotica, Division of Physical Medicine and Rehabilitation, Scientific Institute of Veruno, Veruno, Italy; ^4^Istituti Clinici Scientifici Maugeri Spa SB, IRCCS, Division of Neurological Rehabilitation, Scientific Institute of Veruno, Veruno, Italy; ^5^Istituti Clinici Scientifici Maugeri Spa SB, IRCCS, Centro Studi Attività Motorie, Pavia, Italy; ^6^Department of Public Health, Experimental and Forensic Medicine, University of Pavia, Pavia, Italy

**Keywords:** gait rehabilitation, curved walking, circular treadmill, podokinetic after-rotation, Parkinson’s disease

## Abstract

Training subjects to step-in-place eyes open on a rotating platform while maintaining a fixed body orientation in space [podokinetic stimulation (PKS)] produces a posteffect consisting in inadvertent turning around while stepping-in-place eyes closed [podokinetic after-rotation (PKAR)]. Since the rationale for rehabilitation of curved walking in Parkinson’s disease is not fully known, we tested the hypothesis that repeated PKS favors the production of curved walking in these patients, who are uneasy with turning, even when straight walking is little affected. Fifteen patients participated in 10 training sessions distributed in 3 weeks. Both counterclockwise and clockwise PKS were randomly administered in each session. PKS velocity and duration were gradually increased over sessions. The velocity and duration of the following PKAR were assessed. All patients showed PKAR, which increased progressively in peak velocity and duration. In addition, before and at the end of the treatment, all patients walked overground along linear and circular trajectories. Post-training, the velocity of walking bouts increased, more so for the circular than the linear trajectory. Cadence was not affected. This study has shown that parkinsonian patients learn to produce turning while stepping when faced with appropriate training and that this capacity translates into improved overground curved walking.

## Introduction

Gait disturbances are a critical issue in patients with Parkinson’s disease (PD) (1, 2). Of note, increased risk of falling during transitions (3, 4) and gait asymmetry (5) become more evident when walking along circular rather than linear trajectories (6). During normal curved walking, muscle synergies account not only for the obligatory propulsion but also for the equilibrium constraints connected to body rotation (7–9). Turning involves complex orientation of head, trunk, pelvis, and feet (5, 10–12) and is accompanied by trunk inclination to the inner part of the trajectory to counteract the centrifugal acceleration acting on the walking body (10, 13, 14). Also, motion of the lower limbs is asymmetric, whereby the leg inside the trajectory travels a shorter path than the outside leg (5, 10, 12, 15). These subtasks are normally performed unconsciously, but PD is associated with impaired gait automaticity, such as reduced arm swing, decreased stride length, overall instability (16), and trunk rigidity (17). Not unexpectedly, given the complex coordination and multisensory integration underlying curved walking (17, 18), studies requiring patients with PD to travel both linear and circular pathways have detected additional abnormalities during curved walking (19–23).

Dopaminergic treatment does not always provide substantial improvement of gait (24), and long-term treatment carries increased risk of episodes of freezing of gait (25, 26). Even deep brain stimulation may not provide significant improvement (27). Therefore, gait rehabilitation seems to be a valuable treatment for these patients (28–30), also for coping with changes in walking direction to negotiate corners and avoid obstacles. Thus, rehabilitation of curved walking has been advocated by several investigators (31, 32). In addition to the linear treadmill, the circular treadmill could become a crucial adjunct to the armamentarium of gait rehabilitation, since it induces an aftereffect called podokinetic after-rotation (PKAR), which causes subjects to involuntarily turn around their vertical axis when asked to step-in-place without vision.

While stepping-in-place eyes open on the rotating treadmill, the stance foot repetitively turns relative to the stationary trunk, inducing a novel relationship between foot and trunk position during stepping (33). This stimulation produced by the rotating treadmill has been called podokinetic stimulation (PKS) (34). Most interestingly, following exposure to extended periods of PKS, individuals inadvertently rotate around their vertical axis when stepping eyes closed on a stationary surface, reflecting adaptation of the foot-trunk system. This aftereffect PKAR is induced in healthy adults across a range of stimulus durations and amplitudes (31). The similarity of PKAR to voluntary turning suggests that PKAR may depend on the same neural networks used for voluntary turning (34). Of note, the PKS compels subjects to lift their feet and rotate their legs with an appropriate cadence and amplitude of leg rotation, because subjects are asked to maintain an invariant orientation of their trunk with respect to space. This rhythmic stimulation to step and turn might be relevant regarding treatment, since both rhythmogenesis capacity of the spinal circuitry (35) and stepping in response to perturbations (36) seem to be compromised in patients with PD.

Preliminary data on the potentially positive effect of circular treadmill training in PD have recently appeared (37). The improvement of the velocity of curved walking in these patients after training would possibly rest on the engagement of the neural circuits subserving the complex synergies for turning mentioned above, and on increased strength of the muscles related to turning (38). When administering the PKS to people with PD, Hong et al. (39) found no obvious differences in the PKAR between patients with PD and neurologically healthy older adults (40). On the one hand, failure to detect differences between both groups may have been due to small sample size, or to testing participants on dopaminergic medication that may affect motor adaptation (38), or else combining freezers and non-freezers into a single PD group. On the other, that finding is in favor of an overall capacity of patients with PD to exhibit this type of motor adaptation. This is not unexpected, both because elderly subjects largely retain locomotor adaptability (41) and because basal ganglia damage from degenerative diseases largely leaves adaptation intact (42–45).

Therefore, we hypothesized that having patients with PD stepping-in-place repeatedly on the rotating treadmill would induce adaptation to turns, witnessed by gradual PKAR enhancement. An adapted, dynamic training protocol would be necessary, since patients with PD, tested under different conditions, require a larger number of trials to adapt than age-matched controls (46). In turn, this training would possibly improve overground curved walking. If the ability to adapt to PKS is present in people with PD and does transfer to curved walking, then the rotating platform may potentially serve as a helpful rehabilitative tool for difficulties of turning and curved walking.

The main goal of this study was to determine the features of PKAR in PD patients after PKS. We assumed that PKAR features would be abnormal in PD (39) and that PKAR would increase in velocity and duration across days by incremental training administered with the rotating treadmill. We also tested the hypothesis that the adaptation to PKS would produce an improvement in the velocity of the spontaneous overground walking along circular trajectories.

## Patients and Methods

### Participants

Fifteen parkinsonian patients participated in the study. This sample was chosen based on preliminary data from this laboratory (37). The prospective power calculation had shown that a sample size of 15 would have 80% power to detect a mean difference in gait velocity of 11 cm/s, with a SD of 14 cm/s, using a one-sided paired Student’s *t*-test with alpha of 0.05. Patients were recruited from the local association of PD and from our laboratory database. All patients had a diagnosis of idiopathic PD based on defined criteria (47), and all were on stable dopaminergic medication. They did not change their pharmacological therapy during the study. No patient had orthopedic conditions restricting exercise, or had deep brain stimulation surgery or evidence of dementia (Mini-Mental State Examination <26) (48). All patients could walk independently. Hoehn and Yahr scores (49) ranged between 2 and 2.5. Table 1 provides the patients’ characteristics. All patients were naive to the experimental procedure and all succeeded in performing the tasks.

**Table 1:** **Demographics and clinical details of the patients with Parkinson’s disease**.

Patient	Sex	Clinical phenotypes	Age (years)	Body weight (kg)	Height (cm)	Duration (years)	H&Y	UPDRS III	MMSE
1	M	PIGD	71	99	180	4	2	18	27.4
2	M	TD	76	92	165	14	2.5	26	27.7
3	M	PIGD	82	75	170	8	2.5	14	26.4
4	F	PIGD	69	62	168	11	2.5	15	30
5	M	TD	79	61	169	12	2.5	19	26.7
6	M	TD	76	86	178	4	2	21	26
7	M	PIGD	64	76	162	4	2.5	13	29
8	M	TD	76	76	168	6	2.5	46	30
9	F	PIGD	61	53	165	1	2.5	22	30
10	F	PIGD	67	75	150	7	2.5	15	30
11	F	TD	51	90	173	10	2	17	30
12	M	PIGD	68	70	162	9	2	33	27
13	F	PIGD	65	54	160	2	2.5	37	26
14	M	PIGD	78	100	178	8	2.5	15	28
15	M	PIGD	71	76	180	10	2.5	15	30
Mean			70.3	76.3	168.5	7.3	2.4	21.7	28.3
SD			8.08	14.95	8.42	3.79	0.23	9.74	1.64

*M, male; F, female; H&Y, Hoehn and Yahr scale; UPDRS, Unified Parkinson’s Disease Rating Scale; TD, tremor-dominant; PIGD, postural instability/gait difficulty; MMSE, Mini-Mental State Examination*.

The experiments were performed in accordance with the Declaration of Helsinki. The ethics committee had approved the experimental protocol including the PKS sessions (approval number 806 CEC, protocol GR-2009-1471033). All procedures were carried out with the adequate understanding and written informed consent of each patient.

### Preliminary Assessments

#### Unified Parkinson’s Disease Rating Scale (UPDRS)

Scoring was done once, at baseline evaluation. We used the motor section (III) of the UPDRS (50). This is composed of 14 items, which assess specific disorders such as bradykinesia, rigidity and tremor, balance, and functional mobility (51). Patients were identified as tremor-dominant (TD) or postural instability/gait difficulty (PIGD) based on Stebbins et al. (52) (Table 1). Right vs left differences on the UPDRS III scale were examined. The criteria for excluding asymmetry were those reported in Ref. (53). In most patients, the severity of the motor symptoms was comparable in the right and left sides of the body. Five patients were asymmetric, having a left vs right difference score >5 points (two were more severely affected on the right and three on the left side).

#### Linear and Curved Overground Walking

Each patient underwent gait assessment twice: on the day before the onset of the first training session and on the day following the last training session (as described below). Patients walked at their comfortable speed under three different conditions: linear (LIN) and circular walking, clockwise (CW), and counterclockwise (CCW). The order of these conditions (LIN, CW, and CCW) was randomized across patients. The linear path was obtained by asking the patients to walk down a corridor (2.5 m width). The curved path (1.2 m radius) was drawn with a continuous tape stuck on the floor of a large room (54). Before data acquisition, each patient performed one short trial for each condition (LIN, CW, and CCW) in order to familiarize with the task. Then, patients executed two 20-m length walking trials for each trajectory, making a total of six trials. Patients were instructed to walk looking forward, head erect, without gazing constantly to the tape but walking along it as smoothly as possible. Gait speed, cadence, and stride length were computed.

#### Stepping-in-Place

Before each training session, the patients stepped-in-place for 60 s in the center of the motionless circular treadmill, blindfolded. The purpose of these “control” stepping-in-place trials was to assess that patients had no preferential sense of rotation. Repetition of “control” stepping before each training session ensured that the preceding training session did not affect the orientation in space during stepping-in-place in the following session. Patients were never made aware of their possible rotation and the only indication given was to step-in-place naturally at their own pace. Degrees traveled in the 60 s period, sense of rotation, and cadence were measured.

### PKS Administration and PKAR Evaluation

Patients underwent 10 training sessions, each in a different day. Sessions were repeated two or three times a week in different days over four successive weeks. At the beginning of each PKS trial, the patients entered the rotating platform and put on a security harness (no weight unloading), which they wore during the entire session on the platform. Their arms were free to move, but they were asked not to reach out for support, or use upper limb movements to maintain stability.

#### Structure of the Training Sessions and PKAR Evaluation

**Figure 1:**
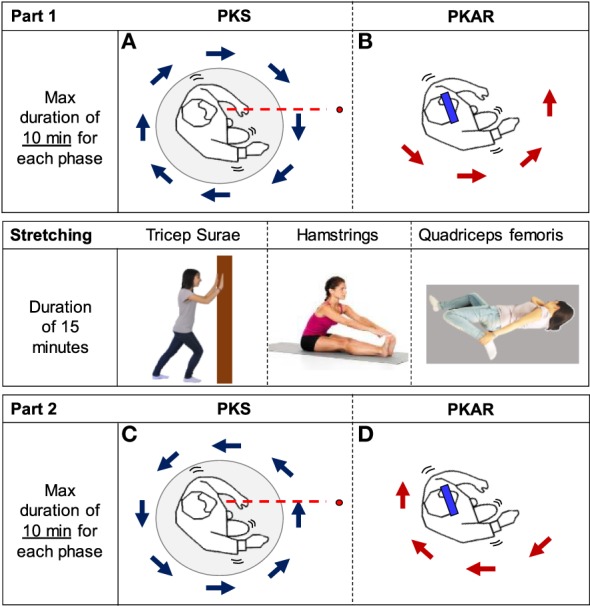
**Scheme of the protocol**. It was composed of the clockwise (CW) podokinetic stimulation (PKS) [**(A)**, PKS] and its aftereffect [**(B)**, podokinetic after-rotation (PKAR)] (Part 1), the Stretching phase, and the CCW PKS **(C)** and its PKAR **(D)** (Part 2). **(A,C)** PKS. **(B,D)** PKAR. In panel **(A)**, a patient steps in place on the center of a platform rotating in CW direction, with the position of the trunk frontal plane roughly perpendicular to a virtual line (red) connecting patient’s eyes to a 3 m distant target (red dot). Panel **(B)** represents the patient, who steps in place blindfolded (blue bar) on the motionless platform, exhibiting the PKAR. During this aftereffect, the patient inadvertently rotates while stepping-in-place, and the direction of body rotation is opposite to the rotation of the platform in **(A)**. Panel **(C)** is similar to panel **(A)**, but the platform rotates in the opposite direction. Panel **(D)** is similar to panel **(B)**, but the patient turns in the opposite direction. The middle panel simply shows images of the “Stretching” phase.

Each training session was divided into three parts (see Figure 1). Part 1 started with the PKS phase that consisted in stepping-in-place eyes open on the center of the platform rotating in CW or CCW direction (Figure 1A). PKS was followed by the PKAR phase that required stepping-in-place with a blindfold on the same platform, motionless (see Figure 1B). PKS had a variable duration, which was set by each individual patient’s tolerance and the judgment of the physiotherapist, who had planned in advance to have patients repeat the same two phases shortly. In any case, the duration of both PKS or PKAR phase did not exceed 600 s, because this was the maximum time allowed based on *a priori* deliberation considering ethical (patient’s fatigue) and practical (total duration of the session) reasons. After Part 1, patients performed a stretching exercise program with the assistance of a physiotherapist that included three bouts of lower limb muscle stretching: quadriceps, hamstring, and calf, bilaterally. The stretching period lasted about 15 min (Figure 1, middle panel). Part 2 was also composed by two phases: PKS in the opposite direction with respect to Part 1 (Figure 1C), followed by PKAR (Figure 1D). The order of the CW and CCW PKS was alternated across patients and across days according to a fixed scheme such that, ultimately, patients performed five sessions of the CW and five sessions of the CCW PKS. These conditions were balanced in order to train turning in both directions in all the patients, with the purpose of providing a functional and ecological exercise appropriate to cope with any changes in walking direction.

#### Setting of the Rotating Platform Features

**Figure 2:**
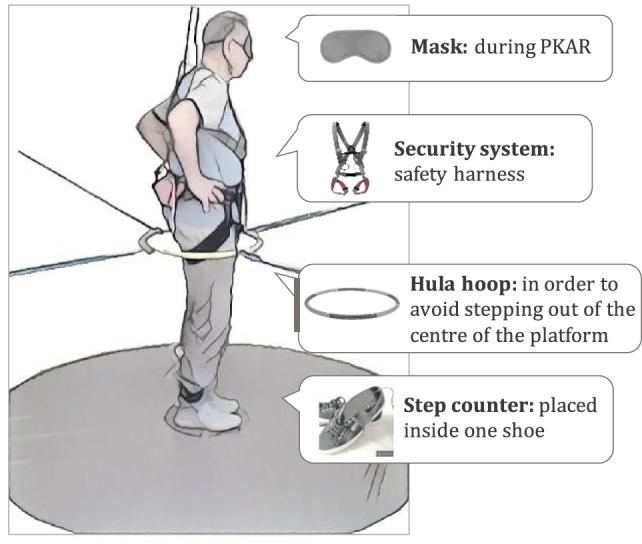
**Drawing of the experimental set showing a patient ready for the training session on the rotating platform**.

The circular platform (Officina Lomazzi, Legnano, Italy) had a radius of 1 m. A brushless motor (220 V) was controlled by a custom-made software written in LabVIEW. For all trials, patients were wearing the following equipment: step counter, mask (only during PKAR), and safety harness. A hula hoop gently maintained the patients in the center of the platform. It was loosely fixed at pelvic height by elastic straps secured to the platform outer railing and prevented patients’ displacement from the platform rotation center while stepping-in-place eyes closed. Lightly touching the hoop with the pelvis occurred from time to time, but gave no cue regarding the body position in space, during either the PKS or the PKAR, as from the patients’ report at the end of the experiments. All patients used the same types of rubber-sole shoes (Superga 2521) (Figure 2).

#### Podokinetic Stimulation

The platform rotated at the angular velocity imposed by the physiotherapist (Figures 1A,C). Angular velocity varied from 0 to 55°/s (maximum), in compliance with the actual capability of each patient to tolerate the imposed rotation. During each training session, the physiotherapist encouraged the patients to maintain a constant orientation of the body in space, facing the target (patient’s eyes were open in this phase), and avoid rotating together with the platform. The physiotherapist adjusted the velocity and duration of the platform rotation with the aim of obtaining a medium-high level of perceived effort, in order to induce a true training effect. Because stepping-in-place requires about the same energy expenditure as walking (55), we considered this task a sort of aerobic exercise and checked exercise intensity by monitoring the rate of perceived exertion by using the Borg scale (56). This was administered every few minutes during each PKS session. In order to try to maintain the same intensity of training (Borg score around 12–13) (57), the platform angular velocity was progressively increased during the same session and across the 10 sessions. Hence, angular velocity and duration were progressively updated according to the actual ability of each patient, as long as the performance improved. The speed of the platform was not increased when it appeared that the patient was not able to maintain the requested body orientation while stepping. PKS was stopped when the Borg score reached 14 points. As said above, we imposed a maximum duration of 600 s for each PKS task, to allow patients performing the two parts of the session (PKS in CW and CCW direction, and corresponding PKAR) in the same day.

#### Podokinetic After-Rotation

At the end of each PKS phase, patients stepped-in-place on the motionless platform, blindfolded (Figures 1B,D). The aftereffect appearing in this period consisted in involuntary turning while stepping-in-place, in the direction opposite to that of the preceding platform rotation. This phase was set to last for a maximum duration of 600 s. The acquisition was stopped before that time in the case of an actual shorter duration of the PKAR (rotation was considered concluded by the absence of turning for at least 10 s), or when the patient declared cramps at the leg muscles or fatigue. All in all, 300 PKAR events were captured in this study: 10 trials after the PKS in the CCW and 10 after the PKS in CW for each of the 15 patients.

#### Step Counter

We used an automatic pedometer. The hardware consisted in an insole with a microswitch connected to a wireless interface in communication with the PC. The software was a mini-APP for Windows developed with Visual Basic 6 SP6 able to count the number of steps and measuring real time and average cadence.

#### Data Acquisition

The features of the PKAR were measured by filming the complete sessions and by the step counter. No metronome was used during the tasks to set cadence. The data from the step counter gave the instantaneous cadence in the different stages of the session, not only for PKAR but also for the “control” stepping trials and for the PKS. At the end of each session, a physiotherapist analyzed the video recordings. When watching the video, she recorded the time taken for the patient to make 45° of rotation. The mean angular speed for every completion of 45° was calculated, until the PKAR finished. Then, the maximum angular velocity reached and total degrees traveled by the patient were noted.

### Statistical Analysis

Results are reported in the tables as mean ± SD and in the text and figures as mean ± SE.

A test for normality (Shapiro–Wilk) was performed prior to statistical comparison of the differences in all recorded variables. The gait characteristics (speed, stride length, and cadence) were normally distributed. Comparison of their mean values at baseline between LIN, CCW, and CW was made by one-way repeated-measure ANOVA tests, separately for speed, stride length, and cadence. Then, to evaluate the effect of the treatment on the spatiotemporal variables of gait (for both linear and curved walking), a repeated-measure ANOVA with 2 within-subject factors was run (direction of rotation: LIN, CCW, and CW; pre- and post-training: T1 and T2), separately for gait speed, cadence, and stride length. For the “control” stepping phases, we used a one-way ANOVA to assess differences in degrees rotated and cadence (both variables were also normally distributed) across the 10 days of training. When the ANOVAs gave a significant result (*p* < 0.05), the *post hoc* Fisher’s test was conducted to assess differences between variables.

The distribution of the variables of PKS (duration and velocity of platform rotation, cadence) and PKAR (duration, degrees covered, maximum angular velocity, cadence) proved to be non-normal. Mean differences between directions (CW and CCW) for all PKS and PKAR variables were evaluated by the Wilcoxon test (separately for each session, all patients collapsed). The Bonferroni correction was applied to compensate for alpha inflation due to multiple comparisons, and *p* < 0.005 was set for statistical significance. Since there was no statistical difference between directions in each of the 10 sessions for all variables, differences in velocity and duration of platform rotation and cadence across days of PKS training were tested by three separate non-parametric statistics (Friedman’s ANOVA) with directions of rotation collapsed. Similarly, Friedman’s ANOVA was conducted for duration, maximum speed, degrees rotated, and cadence of PKAR, across the 10 sessions of training, with directions of rotation collapsed. When Friedman’s ANOVA was significant, the *post hoc* Wilcoxon test was performed for paired comparisons, and the Bonferroni correction applied (statistical significance being set at *p* < 0.001). Likewise, Friedman’s ANOVA was run for comparing the cadence in the three different stepping tasks (“control” stepping, PKS, and PKAR), with patients and days collapsed.

The effect size of the difference between final (T2) and baseline (T1) assessment for speed, cadence, and stride length of linear and curved walking was calculated by using the Cohen method for paired samples. An effect size of 0.20 was considered small, 0.50 medium, and 0.80 large (58).

Statistical analysis was performed using Statistica (StatSoft Inc., Tulsa, OK, USA).

## Results

### Gait Details at Baseline

#### Linear and Curved Walking

Table 2 shows the mean values (15 patients) of speed, cadence, and stride length at the baseline evaluation, for linear walking of 20 m in a hallway LIN and for an equal-length walking in CCW and CW along a circular trajectory of 1.2 m of radius. Speed was different between directions [ANOVA, *F*(2,28) = 79.52; *p* < 0.0001]. *Post hoc* analysis showed that speed was higher in linear than circular trajectories (Fisher’s test, for both directions *p* < 0.0005). Cadence was higher in the linear than curved walking condition [*F*(2,28) = 18.72; *p* = 0.0001], with no difference between CCW and CW directions (*p* = 0.52). Also, stride length was different between walking conditions [*F*(2,28) = 26.718; *p* = 0.0001], longer for linear than circular trajectories (both directions, *p* < 0.0005).

**Table 2:** **Mean gait details of the patients with Parkinson’s disease at the baseline, in linear and both curvilinear trajectory**.

	Speed (m/s)			Cadence (steps/min)			Stride length (m)		
	Linear (LIN)	Counterclockwise (CCW)	Clockwise (CW)	LIN	CCW	CW	LIN	CCW	CW
Mean	1.24	0.92	0.93	123.61	111.23	112.67	1.17	0.98	0.99
SD	0.17	0.16	0.16	11.53	13.04	13.19	0.11	0.10	0.12

#### Stepping-in-Place

**Figure 3:**
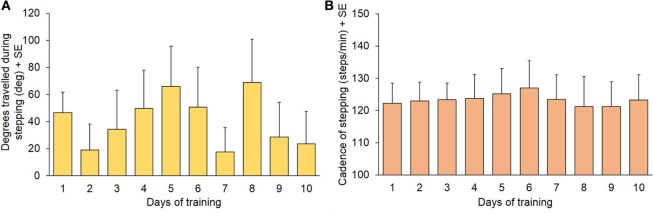
**(A)** Degrees covered during the 60 s “control” stepping phase (mean values of 15 patients). **(B)** Cadence during the same trials (*n* = 14: one patient had episodes of gait hesitation and was not included in the calculation of mean cadence).

Before starting each training session, all patients were required to simply step-in-place blindfolded for 60 s, without any additional instruction *(“control” stepping)*. The physiotherapist recorded the mean cadence and the rotation at the end of the 60 s period, with a positive value if rotation was in CW or a negative value if it was in CCW direction. All patients and both directions collapsed, there was no significant difference in the mean degrees rotated (Figure 3A) during this task across the 10 training session [*F*(9,134) = 0.83; *p* = 0.58]. Figure 3B shows the mean cadence. This was measured in the 14 patients, in which it was possible record the cadence with the step counter (one patient had episodes of hesitation during stepping that prevented the measurement). Cadence remained remarkably constant across sessions [*F*(9,130) = 0.054; *p* = 0.99].

### Podokinetic Stimulation

#### General Features of the Incremental Training

**Figure 4:**
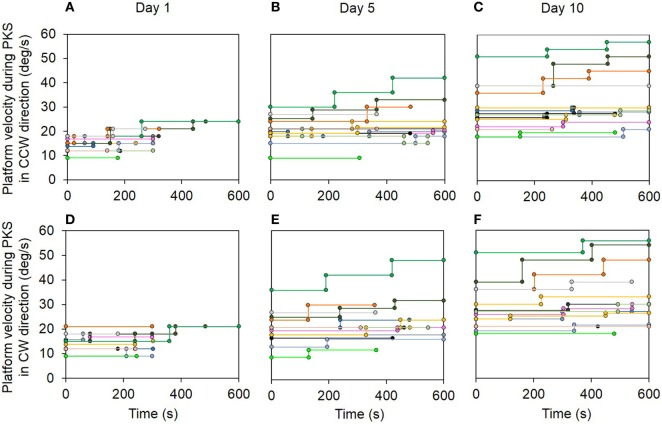
**Top panel shows the actual velocity (ordinate) and duration (abscissa) of the platform rotation during podokinetic stimulation (PKS) at the 1st (A), 5th (B), and 10th day (C) for the counterclockwise (CCW) direction trials**. Bottom panel **(D–F)** represents the same variables at the 1st, 5th, and 10th day for the clockwise (CW) direction trials. Each patient is identified by a different color. Note the overall progression in duration and velocity of rotation of PKS within sessions and across days.

All patients endured stepping on the center of the rotating platform. All were able to sustain higher rotation speeds and longer durations of PKS, as the platform angular velocity and duration was gradually increased both within sessions and through the successive sessions. Overall, the increment in duration and angular velocity proved to be possible in all PKS sessions, regardless of the rotation direction. Figure 4 shows the actual speeds of the rotating platform for all patients during the subsequent training sessions, in both CCW (top row) and CW (bottom row) directions. Each patient performed both CCW and CW trials in each day, randomly across days. The traces show that, within each session, the imposed rotation varied stepwise in velocity (ordinate) and duration (abscissa) depending on the individual patient (identified by different color). In the earlier sessions, the rotation was stopped earlier than in the following sessions. Progressively, velocity and overall duration were increased. At the first day of training, only one patient endured stepping 600 s, and all patients displayed a maximum angular velocity below 25°/s (Figures 4A,D). In the last session, most patients endured 600 s platform rotation, at angular velocities up to 57°/s (Figures 4C,F). All the patients performed the 10 sessions of the training program.

#### Duration, Angular Velocity, and Stepping Cadence of PKS

**Figure 5:**
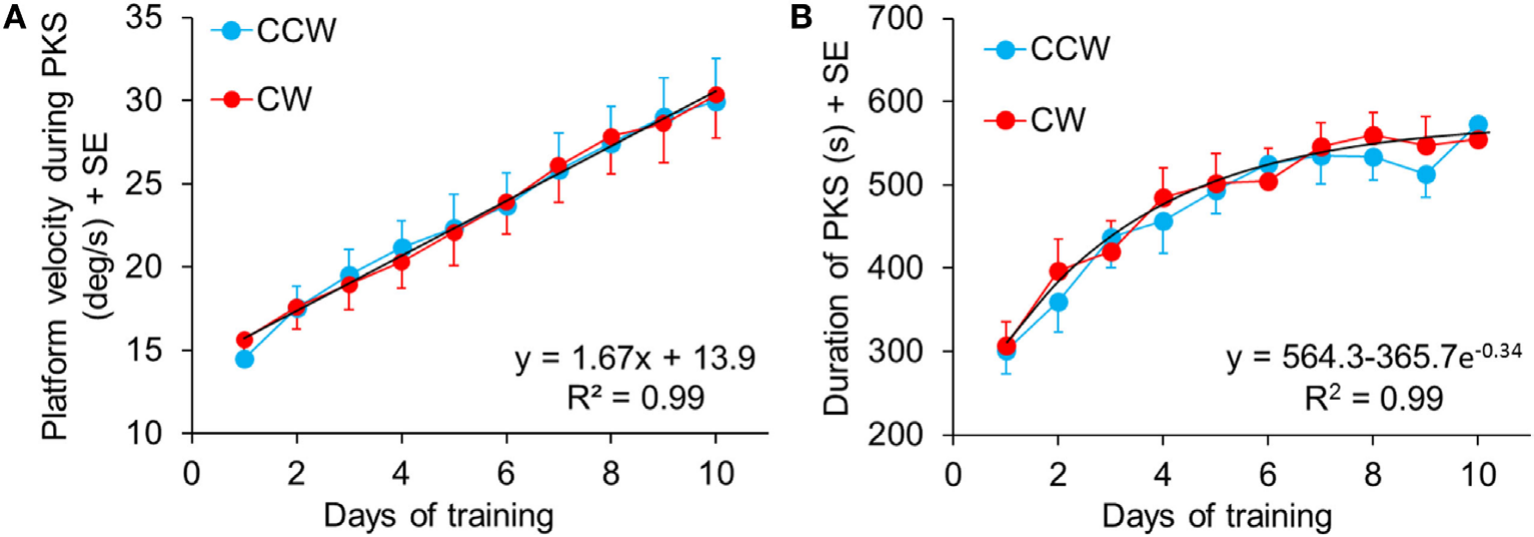
**(A)** Mean speed of the rotating platform during the subsequent training sessions. The blue and red symbols indicate the mean velocity in counterclockwise (CCW) and clockwise (CW) direction, respectively, in each training sessions. The black dashed line shows the regression of the “training” curves (CCW and CW collapsed, since no differences were found between CW and CCW data within the same day). Panel **(B)** shows the mean duration of the podokinetic stimulation (PKS) of the subsequent sessions. The maximum time of training, preliminarily established, was 600 s. No differences were found between CW and CCW within the same day.

Figure 5 summarizes the data showing the progressive increment of angular velocity (Figure 5A) and duration (Figure 5B) of the stepping-in-place during the PKS. The PKS changes over time depended on the patients’ individual capacity of coping with the platform rotation features. In the first session, patients reached, on average, a training duration of 300 s (mean of all patients for both directions). At the last training session, 12 patients completed 600 s PKS (the maximum set duration for PKS), for both directions of rotation; two patients reached a PKS duration of about 480 s in both directions and one patient reached a duration of 600 and 300 s in CCW and CW rotation, respectively. On average, no difference in duration of the platform rotation between directions was found within each training day (Wilcoxon test, *p* > 0.07 for all 10 comparisons). The duration of the platform rotation significantly increased across subsequent days (Friedman’s ANOVA, χ^2^ = 132.1, *p* < 0.0001). The mean durations of platform rotation were significantly longer than at the first day at the 4th, 5th, 6th, 7th, 8th, 9th, and 10th (Wilcoxon *post hoc* test, *p* < 0.0009).

There were no differences in mean angular velocity between directions within each training day (Wilcoxon test, *p* > 0.06 for all 10 comparisons). Angular velocity increased across subsequent days (Friedman’s ANOVA, χ^2^ = 241.08, *p* < 0.0001). Mean angular velocity was 14.5 ± 0.8°/s at the 1st day in CCW and 15.6 ± 0.9°/s in CW. It reached 30.0 ± 2.7°/s at the 10th day and 30.3 ± 2.6°/s, in CCW and CW, respectively. *Post hoc* analysis showed that angular velocities at the 3rd, 4th, 5th, 6th, 7th, 8th, 9th, and 10th day were significantly different from the 1st day (Wilcoxon *post hoc* test, *p* < 0.0007).

Figures 5A,B show velocity and duration of PKS platform rotations across days. There was a good linear fit for velocity (both directions collapsed, *y* = 13.9 + 1.67*x, R^2^ = 0.99, *p* < 0.0001). Visual inspection of the data suggested that the rate of learning to cope with the increasing platform velocity did not change over the course of training. The duration of the PKS as a function of the treatment days showed an initially rapid increase followed by a slower increase, as a consequence of the restriction of PKS duration to a maximum of 600 s, featuring an exponential profile (*y* = 564.3 − 365.7 e^−0.34^, *R*^2^ = 0.99, *p* < 0.0001). Since not all patients endured 600 s even in the last days of training, and no patient received a PKS longer than 600 s, the mean duration values necessarily showed a false ceiling, which became obvious around the last days of training. Taken together, these data suggested that training produced stark adaptation to the features of the PKS and that adaptation continued and strengthened until the end of the training sessions.

**Figure 6:**
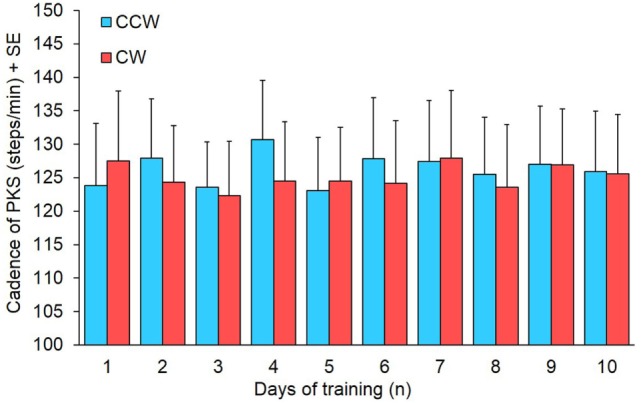
**Mean cadence of 14 subjects in the 10 days of podokinetic stimulation (PKS) training**. Blue and red columns refer to counterclockwise (CCW) and clockwise (CW) direction, respectively. There was no significant difference in cadence between CCW and CW or across days.

Cadence was measured to assess whether the increase in angular velocity of platform rotation during training was accompanied by changes in the stepping rhythm. Mean CW and CCW cadence represented in the bars of Figure 6 were obtained from 14 patients, because in one patient the step counter did not work properly for the presence of episodes of gait hesitation. There was no difference in cadence between directions (Wilcoxon test, *p* > 0.16 for all 10 comparisons) and across training days (Friedman’s ANOVA, χ^2^ = 6.97, *p* = 0.64).

### Podokinetic After-Rotation

#### General Features of the PKAR

**Figure 7:**
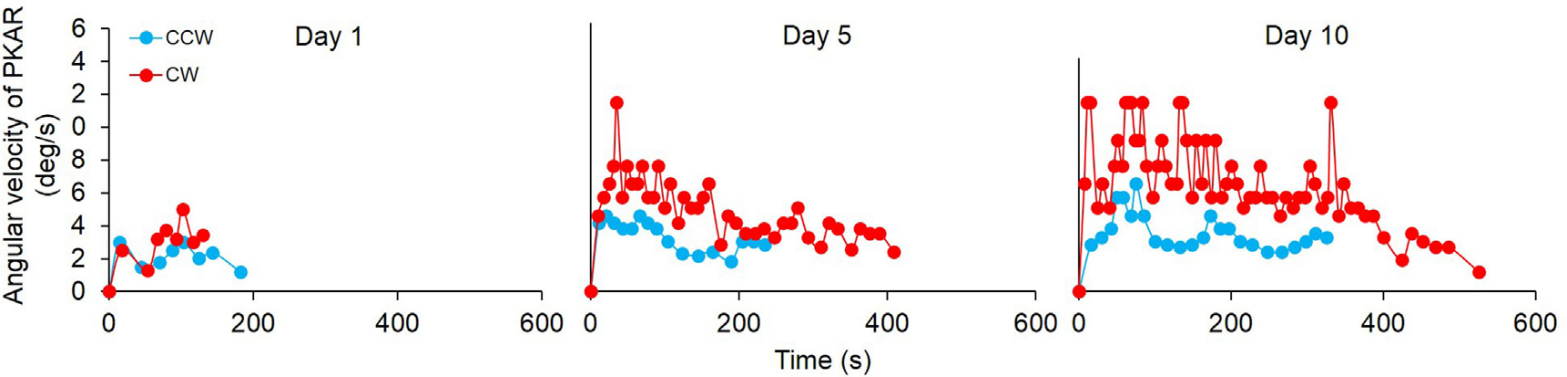
**Time-course of the angular velocity of one representative patient in the podokinetic after-rotation (PKAR) phase, at the 1st, 5th, and 10th day**. Blue values are the PKAR angular velocities after podokinetic stimulation (PKS) in counterclockwise (CCW) direction, red values after PKS in clockwise (CW) direction. There was an increase in both duration and angular velocity across the sessions. In this patient, PKAR was more conspicuous and more variable in CW than CCW direction.

All patients, except one, exhibited a PKAR already at the first session of training. In Figure 7, an example from one patient is reported for the PKAR at the 1st, 5th, and 10th day. Through the successive sessions, there was a trend toward increasing angular velocity and duration of the after-rotation (both directions). In the patient of Figure 7, PKAR rotation faded between 100 and 200 s in the 1st day, while in the last day the effect reached 300 s after CCW PKS and more than 500 s after CW-PKS. PKAR angular velocity also increased across sessions to reach a peak angular velocity of 12°/s at the 10th day. This was about two times higher than the maximum PKAR angular velocity reached at the 1st day. The increasing trend in PKAR angular velocity of this patient reflects the mean trend of all other patients.

#### Duration, Degrees of Rotation, Maximum Angular Velocity, and Cadence of PKAR

**Figure 8:**
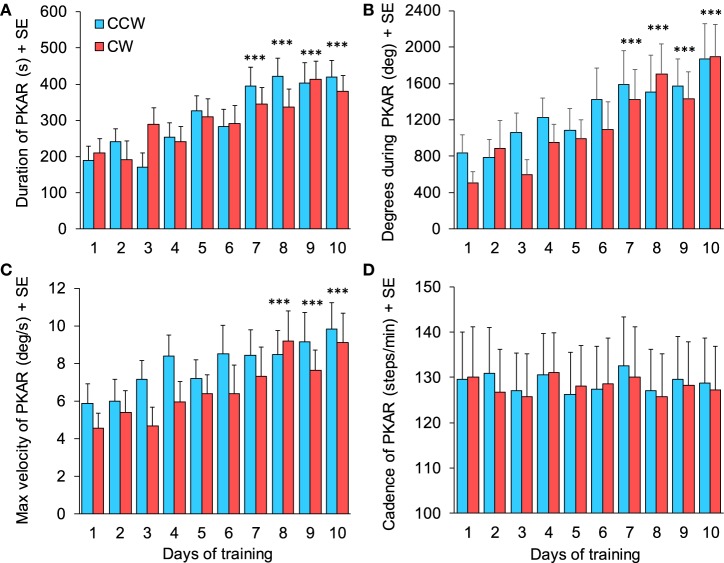
**(A)** Duration of podokinetic after-rotation (PKAR) increased across days for both rotation directions. **(B)** Body rotation (cumulative degrees) covered during the whole PKAR period. The rotation extent regularly increased across days. **(C)** Maximum angular velocity during PKAR also increased steadily. **(D)** Cadence did not change across the 10 sessions of training. Blue and red columns represent PKAR following counterclockwise (CCW) and clockwise (CW) podokinetic stimulation, respectively. There were no significant differences between CCW and CW. Data are means of 15 patients for each of the 10 days of training except for **(D)** (*n* = 14). Asterisks refer to comparison of subsequent days to the first day of training; ***p < 0.0005.

Each of the 15 patients performed 20 trials of PKAR (one trial after CCW and after CW-PKS, repeated for the 10 days of training sessions), for a total of 300 PKAR trials recorded in the cohort. In 18/150 PKAR trials after CCW PKS and in 26/150 trials of PKAR after CW PKS, PKAR lasted until the maximum duration of 600 s: in these cases, rotation while stepping was still obvious at 600 s, but the trials were stopped in accord with the protocol. In general, in approximately 50/300 trials, patients stopped stepping because of cramps or fatigue; these PKAR were kept for the analysis, and possibly contributed to underestimation of the duration of the aftereffect. Figure 8 shows the average values of four main variables of PKAR across days: duration, total angular distance traveled during the stepping-in-place turns, maximum angular velocity achieved, and cadence. For all variables, improvement across sessions was obvious, except for cadence. In each session, the mean duration of PKAR was not different between rotation directions (Wilcoxon test, *p* > 0.26 for all 10 comparisons). Duration increased across the training sessions (both directions collapsed, Friedman’s ANOVA, χ^2^ = 44.68, *p* < 0.0001) (Figure 8A) and the duration at the 7th, 8th, 9th, and 10th days were significantly different from the 1st day (Wilcoxon *post hoc* test, *p* < 0.0001). The number of total degrees traveled was not different between CW and CCW (Wilcoxon test, *p* > 0.13 for all 10 comparisons). It showed an increment across the training sessions (both directions collapsed, Friedman’s ANOVA, χ^2^ = 38.90, *p* < 0.0001) (Figure 8B). The number of degrees traveled at the 8th, 9th, and 10th days was significantly different from the 1st day (Wilcoxon *post hoc* test, *p* < 0.0001).

Maximum angular velocity of PKAR (Figure 8C) increased across days (Friedman’s ANOVA, χ^2^ = 31.68, *p* < 0.0005). Angular velocities at the 8th, 9th, and 10th days were significantly different from the 1st day (Wilcoxon *post hoc* test, *p* < 0.0005). There was no difference between rotation directions (Wilcoxon test, *p* > 0.08 for all 10 comparisons). Cadence (Figure 8D, 14 patients) showed no difference across training days (Friedman’s ANOVA, χ^2^ = 11.29, *p* = 0.26) and between directions (Wilcoxon test, *p* > 0.30 for all 10 comparisons).

### Independence of PKAR Direction Preference and Disease Asymmetry

**Figure 9:**
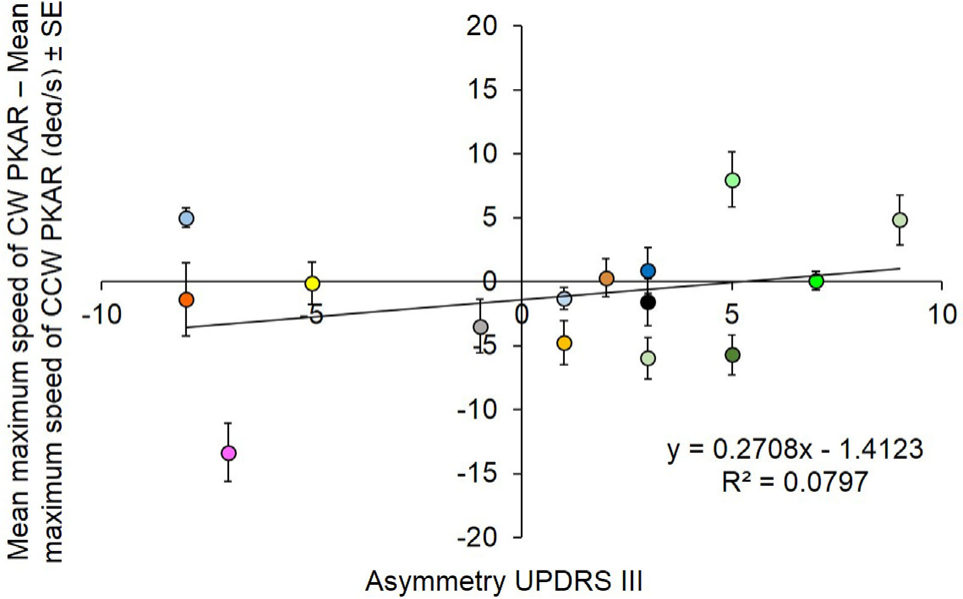
**Each subject is represented with a different color**. The difference between mean maximum speed between clockwise (CW) and counterclockwise (CCW) podokinetic after-rotation (PKAR) is in ordinate. The corresponding asymmetry scores of patients’ clinical severity are reported in abscissa. There is no significant relationship between the two variables.

In order to assess any effect of clinical asymmetry in the disease severity on the susceptibility to the aftereffects of PKS, for each patient the asymmetry of the maximum velocity reached during PKAR was plotted against the asymmetry of the UPDRS III score. Figure 9 summarizes the result, by plotting the relative difference (CW minus CCW) in the maximum velocity reached during PKAR vs the relative side-difference in the severity scores (positive scores correspond to left > right). For each patient, the difference in PKAR velocity is the mean value of the 10 days’ maximum PKAR velocities. No significant relationship was found between the two variables (*R*^2^ = 0.08; *p* = 0.31).

### Relationship between PKAR and PKS

#### Angular Velocity of PKAR Correlates to that of PKS

We correlated the day-by-day values of maximum speed of PKAR to the average speed of PKS, all patients collapsed. Mean speed of PKS of each day was computed as the weighted average, taking into account the duration of the time periods during which a certain velocity was set. Thus, mean speed of PKS was considered to be more representative of the intensity of the PKS phase rather than maximum speed or difference in speed at stop minus start. Maximum speed of PKAR was the maximum speed reached by the patient in the PKAR phase. Maximum speed was chosen instead of mean speed, because it allows to observe the progress of PKAR production without influence of the actual PKAR duration.

**Figure 10:**
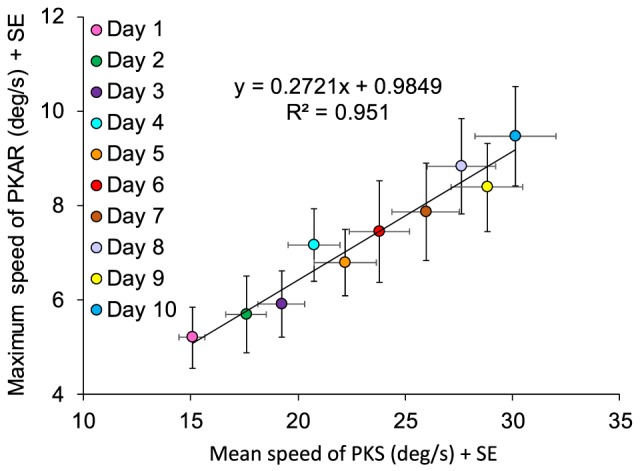
**Data are means of 15 patients in the 10 days of training, mediated for the two directions (counterclockwise and clockwise)**. Maximum speed of podokinetic after-rotation (PKAR) (ordinate) and mean speed of podokinetic stimulation (PKS) (abscissa) are positively correlated.

The correlation between maximum speed of PKAR and mean speed of PKS in the 10 days of training (Figure 10) showed an obvious linear regression (*y* = 0.2721*x* + 0.9849, *R*^2^ = 0.95; *p* < 0.0005). A greater speed in PKS training corresponded to a higher maximum speed of rotation in PKAR. As said above, patients were behaving in the same way in both CCW and CW directions, both in the PKS phase and in the PKAR phase. For this reason, in the figure, we collapsed the data from the two PKS and PKAR directions administered in the same day.

#### Duration of PKAR Correlates to Duration of PKS

**Figure 11:**
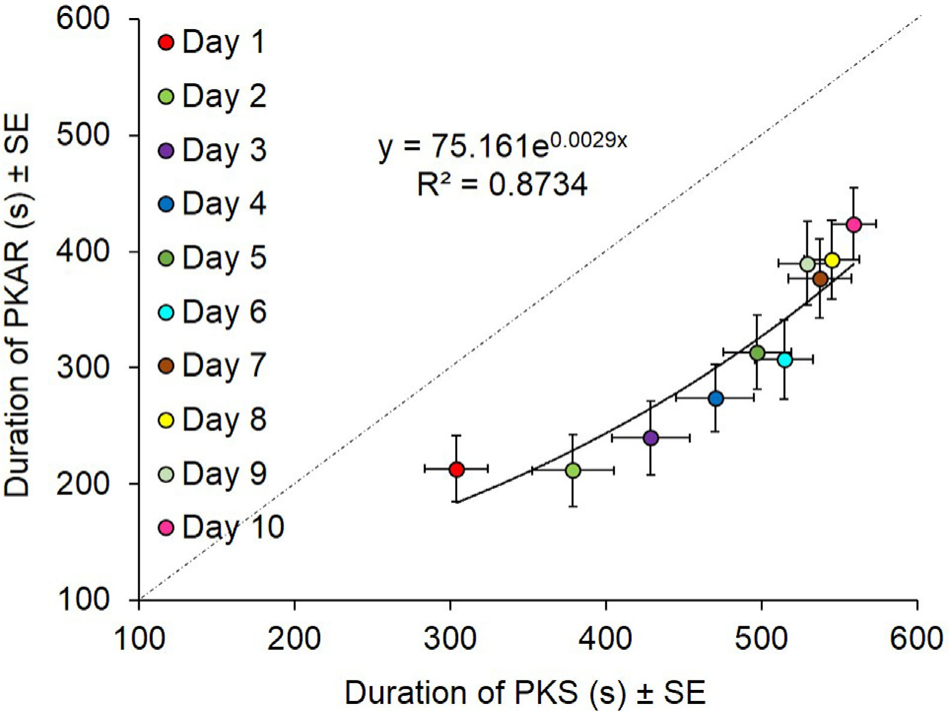
**Data are means of 15 patients in the 10 days of training (counterclockwise and clockwise collapsed)**. Durations of podokinetic stimulation (PKS) and of podokinetic after-rotation (PKAR) are positively correlated. Duration of PKS was higher than the duration of PKAR everyday. Toward the end of the sessions, PKAR could still relatively increase even when the preceding PKS was discontinued on reaching 600 s.

We had imposed 600 s as the limit for the duration of both PKS and PKAR phases. In the case patients who reached that limit in PKS, the platform was turned off. In the case patients who reached that limit in PKAR, recording was also discontinued. PKS and PKAR durations showed a roughly exponential correlation (*y* = 75.161e^0.0029^*^x^, R*^2^ = 0.87; *p* < 0.0005, Figure 11). A greater duration of the PKS corresponded to a greater duration of the PKAR. In general, however, during a single training session, the duration of PKAR was shorter than that of the preceding PKS, as indicated by the distance of the PKAR data points from the identity line. As said above, the rotation direction did not affect the duration of either PKS and PKAR; for this reason, in the figure the data obtained in both directions are averaged.

#### Comparison of Cadence in PKS and PKAR

Cadence was not different across days in all the tasks (see Figures 3B, 6 and 8D). We compared cadence between PKS, PKAR, and the “control” stepping, all days and patients (*n* = 14) collapsed. We found no difference in the mean cadence between the stepping tasks performed in the three different phases (“control” stepping: 122.6 ± 27.1 steps/min; PKS: 123.9 ± 29.9; PKAR: 126.7 ± 33.4) (both directions collapsed, Friedman’s ANOVA, χ^2^ = 1.34, *p* = 0.93).

### Effect of the PKS Training on Linear and Curved Overground Walking

#### Spatiotemporal Variables of Gait

**Figure 12:**
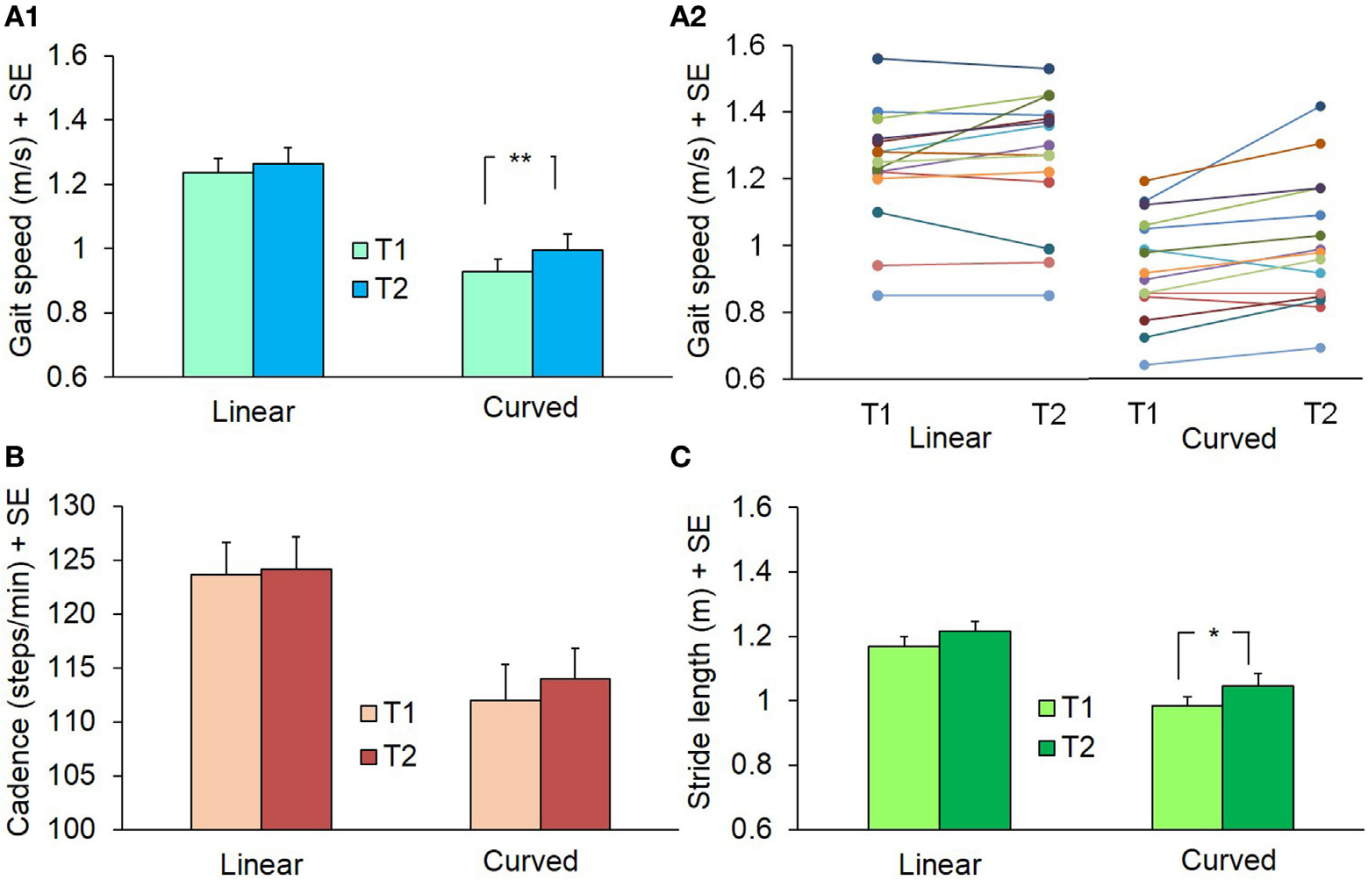
**Mean values of gait speed (A1,A2), cadence (B), and stride length (C) during linear and curved overground walking at the beginning (T1) and at the end (T2) of the 10 training sessions**. Panel **(A2)** represents gait speed in the single subjects (identified by a different color). The curved walking condition data are the grand average of both directions (counterclockwise and clockwise): in all patients but two, velocity increased at T2 (**p < 0.005; *p < 0.05).

As mentioned above, the values of speed, cadence, and stride length did not differ between CCW and CW in the initial assessment of the linear and curved trials of the 15 patients (see Table 2). Figure 12 shows the changes observed prior to (T1) and after completion (T2) of the training sessions. Gait speed was different between baseline and post-training values [ANOVA, *F*(5,70) = 52.69; *p* < 0.0001]. After training, speed of linear gait slightly increased (not significantly so) from 1.24 ± 0.17 to 1.26 ± 0.20 m/s (Fisher’s *post hoc* test, *p* = 0.35), while speed of curved walking increased significantly from 0.93 ± 0.16 to 1.0 ± 0.19 m/s (CW, *p* < 0.05) and from 0.92 ± 0.16 to 1.0 ± 0.20 m/s (CCW, *p* < 0.05). Cadence was slightly higher in linear than circular trajectories [*F*(5,70) = 13.46; *p* < 0.0001], but no changes were found between T1 and T2 (*p* = 0.38). Stride length was different between T1 and T2 [*F*(5,70) = 21.84; *p* < 0.0001]. Stride length varied slightly in the linear walking condition, from 1.17 ± 0.11 to 1.22 ± 0.11 m (*p* = 0.28), but increased from 0.99 ± 0.12 to 1.04 ± 0.14 m in CW (*p* < 0.05) and from 0.94 ± 0.10 to 1.05 ± 0.15 m in CCW condition (*p* < 0.05). So, the training-produced changes in speed and stride length of overground gait were larger in curved walking. The effect size (T2−T1) for gait speed and stride length was small (0.15) in the linear walking condition, and moderate (0.47) in the curved walking conditions (CW and CCW collapsed).

#### Relation between Intensity of PKS and Curved Walking Improvement

**Figure 13:**
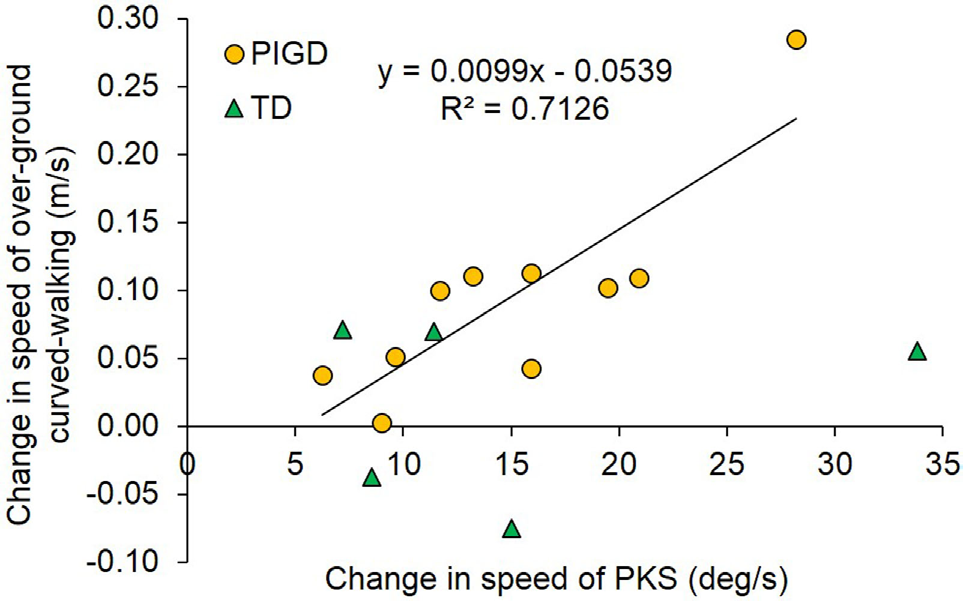
**The plot shows the correlation between the pre-training/post-training change in speed of overground curved walking (ordinate) and the change in podokinetic stimulation (PKS) angular velocity from the first to the last session (abscissa)**. The regression line was drawn only for the postural instability/gait difficulty (PIGD) group (*n* = 10), represented by yellow circles. The tremor-dominant (TD) patients represented by green triangles (*n* = 5) were excluded from the regression.

Figure 13 shows that, across patients, the increment in speed of overground walking along the circular trajectory between baseline (T1) and final assessment (T2) was related to the increment of PKS intensity. For each patient, the change in imposed angular rotation velocity of PKS was obtained by subtraction of mean velocity in the first session from that in the last session. The change in overground curved walking speed was the result of the subtraction of T1 from T2 values. Overall, it appeared that the angular velocity of PKS was often accompanied by a roughly proportional change in speed of overground curved walking. The linear regression across all data points did not reach significance (*R*^2^ = 0.23; *p* = 0.07, not shown in figure). However, the regression became largely significant (*R*^2^ = 0.71; *p* = 0.002) when the five patients clinically defined “tremor-dominant” (green triangles) were excluded from the analysis. A best-fit line drawn through the green triangles would be almost flat, suggesting that even intense PKS would not affect speed of walking along circular trajectories in “tremor-dominant” patients, contrary to “postural instability/gait difficulty” patients.

## Discussion

Many people with PD exhibit difficulty when turning while walking [see Ref. (59) for a recent short review]. Several studies have dealt with this issue and found that whole-body coordination when turning is particularly deranged in patients with PD (6, 60–62). Hong and Earhart (32) have shown that only certain aspects of impaired turning are responsive to medication and encouraged rehabilitative approaches to address turning. Preliminary findings (63) have shown that a short training session consisting in walking along the border of a circular treadmill was sufficient to decrease freezing episodes in parkinsonian patients. We decided to train optimally medicated patients as suggested by Roemmich et al. (45), who conjectured that dopaminergic pathways enhance aftereffect storage in walking rehabilitation.

Here, we advanced the approach described in Ref. (63) and embarked on a complex study aimed at verifying the hypothesis that parkinsonian patients can improve their production of curved walking by learning the basic features of turning while stepping and can transfer their newly acquired pattern to overground walking along a circular trajectory. To this aim, we exploited a paradigm described long time ago in Ref. (31). After prolonged stepping-in-place on the center of a rotating platform, subjects asked to walk normally on firm floor straight-ahead without vision unknowingly generated a curved path. Moreover, when subjects were asked to step-in-place without vision after having stepped on the rotating platform for a prolonged period, they continued to rotate around their vertical axis for a while after the stop of the platform (64–66).

This aftereffect had been named PKAR (33). It is evidence that the PKS induced by the rotating platform prompts the nervous system to produce body rotation during stepping. Interestingly, the overall pattern of body turning (with respect to platform) while stepping during the PKS is superimposable to voluntary turning while stepping and is in turn superimposable to the inadvertent turning during the PKAR (33). The similarity of PKAR to voluntary turning and to its aftereffect suggests that PKAR may depend on the same neural networks responsible for voluntary turning. These neural networks are those that control intra- and extra-rotation of the trunk on the legs, therefore those active during the turning phases of walking normally occurring in the activities of daily living.

### PKAR Is Present in PD Patients and Is Enhanced by Circular Treadmill Training

We have shown that patients with PD were able to exhibit a clear-cut PKAR. Compared to normal young subjects (33, 64), their PKAR was initially erratic, short-lasting, and attained a pretty low rotation velocity, in spite of the patients being medicated. This is in keeping with recent findings by Nemanich and Earhart (40), who have shown that patients with PD, off-medication, exhibited smaller PKAR peak velocities compared to age-matched healthy people. Not unexpectedly, among their patients, those with freezing of gait had the smallest PKAR.

In our patients, however, with the repetition of the training sessions (accompanied by the systematic increase of platform angular velocity), the aftereffect increased in both duration and velocity, so that improvement in the turning velocity during PKAR progressively and gradually increased from the 1st to the 10th day of training. The increasing intensity in the PKS training appears to have been appropriately dosed in each patient. In our case, the improvement in PKAR velocity might have probably approached the turning velocity found previously in a cohort of healthy subjects (33), if each PKS session was not limited to 600 s duration and the maximal mean velocity of the rotating platform was not limited to 57°/s.

Overall, the stepping cadence was remarkably constant during all tasks that were included in the study. Cadence was not different among “control” stepping on the motionless platform performed at the beginning of each day’s training and stepping during the PKS on the platform rotating either CW or CCW, and stepping during the following PKARs. These findings are in keeping with those recently obtained in a population of young subjects (33). Moreover, cadence showed no changes across the training sessions. Cadence appears to be a remarkably constant variable, so that no changes in body angular velocity during PKS or PKAR can be attributed to “improvement” in the frequency of the rhythmic output of the central pattern generator of stepping. Also in other studies, cadence appears to be the variable least affected by gait training (28, 67–69).

In our hands, the parkinsonian patients definitely showed a progressive adaptation of their motor output in response to stepping-in-place on the rotating platform (39, 45). Patients learned to step at higher rotation velocities as a function of the increase in the velocity of the platform rotation. Since cadence was excluded as a cause of increased angular velocity, the circular treadmill training did necessarily increase their capacity to improve activation of the pelvic muscles responsible for leg intra- and extra-rotation and produce a progressively larger foot intra- and extra-rotation. PKAR also increased in duration, so that in some patients PKAR was still detectable when the platform rotation had reached the predetermined maximal duration (600 s). Interestingly, the ratio of the velocity of the PKAR to that of the PKS was consistently close to 1/3 in the different subsequent sessions, in spite of the progressive increase in PKS angular velocity. It was previously shown in normal subjects that for rotating treadmill velocities ranging from 10 to 60°/s, a normal PKAR response has a peak velocity approximately 1/3 the velocity of the treadmill (33, 66).

### Potential Mechanisms Responsible for Stepping Improvement

The rotating treadmill must trigger a collection of afferent information, at the same time as it forces the buildup of descending commands, conveyed across space and time to the body muscles responsible for turning. Most likely, these events require attention and control by the participants, particularly during the initial stepping movements on the rotating platform when the appropriate rhythm and leg rotation amplitude is being defined. Then, the repetition of the stepping and leg rotation tasks becomes automatic, to the point that, when stepping continues on the motionless platform, subjects exhibit a fully involuntary and unperceived turning that can last several minutes. In this light, the administered training seems to be more than appropriate for patients known to suffer from both somatosensory abnormalities (70) and distal motor deficits (71). The process whereby this automaticity and involuntariness was reached required different time intervals across the patients, and probably was not complete in all patients at the end of the treatment. In this regard, our analysis fell short of identifying potential sources of interpatient variability.

Both afferent input and central circuits likely cooperate in sustaining stepping and turning [see Ref. (33) for a brief discussion]. Sort of a “Kohnstamm phenomenon” would ensue, similar to the production of a curved locomotion trajectory after an intense voluntary trunk rotation effort (72). While we are not in the position of drawing on this point, we would remind that spindle feedback, enhanced by both descending fusimotor drive and continuous, rhythmic lower-body spindle activity would be one factor responsible for building-up adaptation during PKS and for sustaining rotation during the PKAR in these patients. Other lines of research have shown that muscle contraction and vibration (both adequate stimuli for spindles) can produce long-lasting effects on central circuits controlling the perception of self-motion (73). If the continuous afferent discharge from the spindle plays a major role in adaptation, then the duration of this long PKAR imitates the action of neck muscle contraction and vibration on perception of whole-body rotation around the body axis (73, 74). Repeated PKS would enable the spindle input to gradually access the central pattern generators (75), overcoming rigidity (76, 77) and help normalizing the production of coordinated pelvis and leg muscle activity. On top of that, one would also consider that PKS requires a non-negligible mental/motor effort. On the one hand, motor imagery *per se* can increase spinal reflex excitability (78, 79), on the other, it is known from a normal elderly population study that motor effort improves muscle strength and descending command, even with training at low exercise intensity (80).

The role of descending influences on leg muscle reflexes has been addressed in PD patients (81). With PKS, these reflex modulations might contribute to the enhancement of the stepping pattern as training continues. Solopova et al. (82) showed that MEPs and H-reflex elicited in the lower limb muscles were significantly smaller during vibration-induced than voluntary cyclic leg movements. Their findings highlight the facilitatory effect of voluntary control of stepping on spinal motor circuits and support the idea of active engagement of supraspinal motor areas in developing central pattern generator-modulating strategies [e.g., Ref. (83), for a study during normal development]. The fact that patients are forced to voluntarily step and cyclically rotate their legs and feet during the imposed PKS may train the supraspinal circuits responsible for the task (84, 85). More pathways might contribute, like cutaneous reflexes (84), present and effective in PD (86). These reflexes might be further enhanced and incorporated into a functional pattern as long as PKS training continues. Training-induced increased strength of the muscles producing rotation of the trunk on the stepping foot would also contribute to enhanced angular velocity of podokinetic adaptation (38). It is remarkable here, however, that PKS can produce PKAR and increase its duration over time in the initial sessions already (even if repetition further improves PKAR), emphasizing nervous adaptation rather than increased muscle force.

### Does PKAR Transfer to Overground Curved Walking?

At the end of the training period, gait speed increased during both linear and curved paths. The mean changes were of 2 and 7 cm/s (i.e., about 2 and 8% compared to baseline), respectively. These changes are due to increased stride length (significant for curved but not linear walking), since cadence did not change significantly. It seems therefore that PKS by the rotating platform increases the performance of walking trajectories in these PD patients, without negatively affecting linear walking. On note, the values of the observed increments are larger than the changes considered significant for a PD population in a recent Cochrane revision (87). Most likely, the progressive increment in PKS intensity was responsible for the improvement. Resistance training seems to be superior to non-resistance training or no intervention on strength and physical function on muscle, even if it may not be as effective for improving gait as it is for balance (88). This might depend on the target of the intervention. Targeting curved walking seems to be appropriate, because it can indirectly favor everyday locomotion, in which good control of stepping *and* balance is critical.

We note that PKS is certainly effective in favoring the active extra-rotation of the trunk on the stance foot (in the direction of the induced body rotation during stepping) and the consecutive intra-rotation of the swing leg with regard to trunk, observed during the PKAR. However, stepping at the center of the rotating platform does not expressly exercise the mediolateral control of equilibrium, crucial during walking, and turning (7, 14, 89). This might explain the ultimately not too large increase in walking velocity post-training along the overground curved path in this group of patients (90). Not unlikely, associating sessions of stepping on the center with sessions of walking on the border of the circular treadmill might produce even larger curved walking improvement.

### Limitations

This is the first study that examines the ability of PD patients to improve walking along a curved path in response to long-term stepping adaptation on a rotating platform without external cues. However, several limitations should be highlighted, and new experiments suggested.

The sample size of the parkinsonian patients’ population is limited. Perhaps, with a larger cohort, we could have traced the differences in improvement exerted by PKS to individual characteristics, like among others age, medication, PD severity scoring, rate of disease progression (91), in addition to clinical phenotype (TD vs PIGD). Further, our patients had largely different levels of physical fitness, but these were not assessed by appropriate cardiopulmonary exercise testing. Moreover, the absence of a control population of age-matched healthy subjects, or of patients affected by turning difficulties of a different nature (92), does not allow to assess whether the positive effects obtained by PKS training are specific to parkinsonian patients.

We would also note that the PKS training was limited to 10 sessions and 600 s per session. Progressive-resistance exercises are known to improve motor performances in parkinsonian patients in the long run (93). Hence, it is not unlikely that more numerous, longer periods of circular treadmill rotation and higher angular velocities of imposed rotation would have produced larger improvements in overground circular walking (94). Previous studies have shown that angular velocity of treadmill rotation during PKS influences PKAR velocity (64). PKAR velocity is also dependent on the amount of time spent on the treadmill, with longer PKS durations resulting in higher PKAR velocity (66). Actually, the protocol employed here did not allow to assess whether a ceiling effect would be reached in PKAR. Furthermore, no reiterated assessment of walking velocity along circular trajectories has been made in the weeks/months following the rotating treadmill training. This prevents arguing on the duration of the improvement.

## Conclusion

Training by stepping in the center of a rotating platform, implying continuous and coordinated intra- and extra-rotation of the lower limbs, is performed relatively easily by parkinsonian patients. Repetition of such training sessions definitely improves the capacity of coping with the task. Besides, a true adaptation to the task ensues, attested by the progressive development of an aftereffect consisting in an involuntary rotation while stepping on firm ground. Moreover, the velocity of overground walking along a curved path increases after the training period. On the one hand, the new finding of a progressively increasing posteffect induced by the rotating treadmill training (the inadvertent “podokinetic adaption”) speaks for a definite capacity of learning to turn while stepping in parkinsonian patients (95). On the other, the rotating platform is proposed as a new tool for rehabilitation of curved walking in PD patients, a critical task in these patients, hardly addressed by current rehabilitation treatments (30, 96). Possible advantages of the rotating platform with respect to overground curved walking training may depend on the possibility that this type of exercise might contain subtasks comparable to those contained in dance exercise, which has been used with success in rehabilitation training in parkinsonian patients (97, 98). Further, and importantly so as far as the rehabilitation design is concerned, the rotating platform offers continuous stimulation, standardized training, safety setting, and no provocation of dizziness.

In order to recommend a comprehensive approach to the issue of curved walking in parkinsonian patients, the results of further experiments would be necessary, in which patients would be asked to also walk along the edge of the rotating treadmill. This latter task does not promote much the rotation of the legs relative to pelvis, but challenges the control of balance along the frontal plane (99) and the production of the appropriate centripetal force for a given angular velocity (14). This second option of the use of the circular treadmill, and the potential advantage of combining stepping at the center and at the edge of the rotating platform, will be tested in a following investigation.

## Author Contributions

MS, MGo, and AN designed the study; AN and FP performed the neurological evaluations; MGo, MGi, and AT performed the experiments; MGi, AT, and MC collected and processed the data; MS, MGo, and MGi wrote the manuscript; AN revised the manuscript.

## Conflict of Interest Statement

The authors declare that the research was conducted in the absence of any commercial or financial relationships that could be construed as a potential conflict of interest.
